# Identification of Candidate Blood mRNA Biomarkers in Intracerebral Hemorrhage Using Integrated Microarray and Weighted Gene Co-expression Network Analysis

**DOI:** 10.3389/fgene.2021.707713

**Published:** 2021-07-19

**Authors:** Feng Jin, Lei Li, Yuehan Hao, Ling Tang, Yuye Wang, Zhiyi He

**Affiliations:** Department of Neurology, The First Affiliated Hospital of China Medical University, Shenyang, China

**Keywords:** intracerebral hemorrhage, candidate blood messenger RNA biomarkers, competitive endogenous RNA microarray, differentially expressed genes, weighted gene co-expression network analysis, Gene Expression Omnibus

## Abstract

**Purpose:**

Intracerebral hemorrhage (ICH) is a serious public health hazard due to its high morbidity, disability, and mortality. Currently, the exact molecular mechanisms of ICH are unknown. We tried to identify the ICH-related candidate blood messenger RNA (mRNA) biomarkers by microarray analysis and weighted gene co-expression network analysis (WGCNA).

**Materials and Methods:**

We collected the blood samples from patients with ICH (*n* = 4) and from vascular risk factor (VRF) controls (*n* = 4) and analyzed the mRNA expression profiles by competitive endogenous RNA (ceRNA) microarray. Differentially expressed genes (DEGs) were identified and then a weighted gene co-expression network was constructed. Modules with clinical significance were distinguished. Then, we downloaded two Gene Expression Omnibus (GEO) datasets (GSE24265 and GSE125512). Candidate mRNAs were identified by taking the intersection of the DEGs in our microarray, the interesting genes in the key module, and the DEGs in GSE24265. Functional analysis involving Gene Ontology (GO) and Kyoto Encyclopedia of Genes and Genomes (KEGG) and construction of a protein–protein interaction (PPI) network were conducted.

**Results:**

A total of 340 DEGs in our microarray were identified between the ICH group and the control group. Among the eight gene modules established by WGCNA, the yellow module containing 191 genes was the most strongly associated with ICH. Four candidate mRNAs (C3AR1, PAWR, ARNTL2, and LDLRAD4) were identified. In the early stage of ICH (within 24 h), C3AR1, PAWR, and ARNTL2 were highly expressed in the perihematomal tissue, but with low expressions in peripheral blood; in the late stage (72 h after the first blood draw), an obvious upward trend of C3AR1 and PAWR in peripheral blood was seen. Functional analysis showed that candidate mRNAs were concerned with multiple pathways, such as the Wnt signaling pathway and calcium signaling pathway. They might affect the process of ICH through neuroinflammation, cell apoptosis, and pyroptosis.

**Conclusion:**

We identified four candidate blood mRNAs (C3AR1, PAWR, ARNTL2, and LDLRAD4) related to ICH. They showed different expression patterns in peripheral blood and perihematomal tissues and changed with time. They might play important roles in ICH through neuroinflammation, cell apoptosis, and pyroptosis and might shed new light to novel biomarkers or therapeutic targets in ICH.

## Introduction

Intracerebral hemorrhage (ICH) is a type of serious cerebrovascular disease with high morbidity, disability, and mortality all over the world (Collaborators, 2019). Patients with ICH usually have poor prognosis and limited treatment options. Neurological dysfunction in patients with ICH is mainly caused by the degeneration, necrosis, or apoptosis of nerve cells of the perihematomal areas in the brain. All these pathological processes are closely related to gene expression and regulation (Qureshi et al., 2009; Aronowski and Zhao, 2011).

Efforts have been made by a few researchers on the gene expression profile in ICH. The gene expression profiles of intrastriatal blood infusion ICH model rats were assessed using microarrays, and 369 differentially expressed genes (DEGs) were found as compared with saline-injected controls (Lu et al., 2006). By microarray analysis, 468 DEGs were identified in the perihematomal areas of brain samples from patients with ICH (Rosell et al., 2011). Nevertheless, the most original dataset of gene expression in ICH is not publicly available. We have searched the Gene Expression Omnibus (GEO) database, one of the most famous microarray and high-throughput gene expression databases in the world, but only two datasets about human ICH were found. One was an RNA sequencing (RNA-Seq) dataset (GSE125512) of peripheral blood samples taken from patients with ICH provided by Kyle B. Walsh, an American researcher, and the other was a microarray dataset (GSE24265) of brain tissue provided by Anna Rosell, the above-mentioned Spanish researcher (Rosell et al., 2011; Walsh et al., 2019).

Bioinformatics, as an emerging subject, plays a more and more important role in promoting the study of gene expression and regulation of human diseases. Weighted gene co-expression network analysis (WGCNA) is a powerful systems biology method for analyzing the gene expression patterns in multiple samples (Langfelder and Horvath, 2008). Correlated genes are clustered into various modules represented by different colors, making it easier to find the relationship between modules to external sample traits, such as clinical diseases. Genes with the most connectivity in the module can be defined as hub genes as they usually play key roles in biological processes (Langfelder et al., 2008).

For many years, people have been trying to discover the biomarkers in peripheral blood to access pathological processes or predict outcomes for ICH (Kamtchum-Tatuene and Jickling, 2019). Changes of the transcriptome level in peripheral blood are of great significance for the study of the molecular mechanisms of diseases (Sharp et al., 2011). These changes include messenger RNA (mRNA) and non-coding RNA, such as microRNA or long non-coding RNA (lncRNA). Studies have shown that blood mRNA can be used as a biomarker for the classification of ischemic stroke and the differential diagnosis of transient ischemic attack (TIA) (Jickling et al., 2010, 2012). However, there is no report that the mRNA expression in the peripheral blood could be used as a biomarker for ICH.

The objective of our study was to identify the candidate blood mRNA biomarkers involved in ICH. We conducted competitive endogenous RNA (ceRNA) microarray analysis of peripheral blood from patients with ICH and then utilized integrated bioinformatics methods, including WGCNA, to identify the candidate mRNAs and verify their expression patterns in public datasets (GSE24265 and GSE125512). Finally, Gene Ontology (GO) terms, Kyoto Encyclopedia of Genes and Genomes (KEGG) pathway enrichment analysis, and gene set enrichment analysis (GSEA) were carried out to illustrate the possible mechanisms of candidate mRNAs in the process of ICH.

## Materials and Methods

### Study Subjects

Four male subjects with hypertensive spontaneous intracerebral hemorrhage (ICH group, *n* = 4) and four male vascular risk factor (VRF) controls (control group, *n* = 4) were recruited from the First Affiliated Hospital of China Medical University. Patients were suitable for entry if they presented within 24 h of onset with a primary basal ganglia hemorrhage (putamen or thalamus). ICH was diagnosed by neurologists based on acute symptomatic manifestations and CT brain scan. Vascular risk factor controls (VRFCs) were subjects diagnosed with hypertension, but did not have prior ICH, ischemic stroke, or TIA. In addition, VRFCs were matched to ICH patients for age, gender, diabetes mellitus, hyperlipidemia, smoking history, and coronary artery disease. The procedures were approved by the ethics committee of the First Affiliated Hospital of China Medical University (approval no. 2012-38-1), and written informed consent was obtained from participants or their proxy. ICH patients were excluded if they had a secondary brain hemorrhage due to brain trauma, tumor, vascular malformation, or anticoagulant use.

### Blood Sample Collection and Microarray Data Processing

Blood was collected from ICH patients within 24 h of disease onset and from control subjects at study entry. Serum was aspirated by centrifugation at 1,200 × *g* for 10 min at 20°C in a mini-41C centrifuge. Total RNA was extracted using TRIzol reagent. RNA was purified by QIAGEN RNeasy Mini Kit (QIAGEN, Hilden, Germany) and converted to complementary DNA (cDNA) using the Agilent RNA Spike-In Kit and cDNA Master Mix (Agilent Technologies, Santa Clara, CA, United States), then labeled using Transcription Master Mix. The concentration of cRNA was determined by spectrophotometry. The samples required *A*_260_/*A*_280_ absorbance ratios between 1.9 and 2.1. Then, the product was hybridized to Agilent LC Human ceRNA array 4 × 180 K (Agilent Technologies) according to the manufacturer’s instructions. The original image was scanned using Agilent Scanner G5761A (Agilent Technologies). Going through the data processing by Feature Extraction software (version 12.0.3.1; Agilent) and Genespring software (version 14.8; Agilent), we obtained the gene expression matrix. The ceRNA microarray matrix contained the expression matrices of mRNAs and non-coding RNAs. The expression matrix of mRNAs was retained for further analysis.

### Identification of Differentially Expressed Genes

Normalization of the expression data and screening of DEGs were performed using the “limma” package in R language (version 3.6.3) (Ritchie et al., 2015). The statistical methods to calculate the *p*-value and false discovery rate (FDR) were the *t*-test and the Benjamini–Hochberg method, respectively. Using a threshold of *p* < 0.05 and the absolute value of fold change > 1.5, the DEGs in our microarray study with statistically significant differences between the ICH group and controls were identified. Volcano plots and hierarchical clustering heatmaps were run using the R package “ggplot 2.”

### GO and Pathway Enrichment Analysis of DEGs

We used the R package called “clusterProfiler” to carry out the GO terms and KEGG pathway enrichment analysis (Yu et al., 2012). The significance threshold was set to *p* < 0.05. The enrichment results of GO biological process (BP), cellular component (CC), and molecular function (MF) of DEGs were obtained and visualized using the R package “GOplot.”

### Weighted Gene Co-expression Network Construction

The WGCNA package in R was utilized to construct a gene co-expression network (Langfelder and Horvath, 2008). Firstly, the top 5,000 ranked genes were screened out by median absolute deviation (MAD). Secondly, we found a soft-thresholding power β with the help of the “pickSoftThreshold” function. Afterward, we transformed the gene expression matrix into a topological overlap matrix (TOM), and then the genes were separated into multiple gene modules. Module–trait relationships and gene significance (GS) across modules were illustrated. The module with the highest value of GS was selected as the key module. In the key module, genes whose module membership (MM) > 0.9 and GS > 0.3 were defined as interesting genes.

### Data Acquisition of Validation Datasets

By searching the GEO database^1^ with the keyword “intracerebral hemorrhage,” two datasets (GSE24265 and GSE125512) were chosen as the validation datasets. GSE24265, including 12 brain samples from four deceased patients with ICH, was based on the GPL570 platform (Human Genome U133 Plus 2.0 Array; Affymetrix Inc., Santa Clara, CA, United States). Tissue samples from the perihematomal areas were defined as the PH group, while those from the contralateral gray and white matter were defined as the normal brain tissue (NBT) group. GSE125512 contained 22 blood samples from 11 patients with ICH. The peripheral blood of patients was collected in two time periods: Within 24 h of symptom onset and 72 h following the first blood draw. GSE125512 was based on the GPL15433 platform (Illumina HiSeq 1000; Illumina, San Diego, CA, United States).

### Identification and Verification of Candidate mRNAs

DEGs between perihematomal tissues and normal brain tissues were screened out through differential analysis by the “limma” package in the dataset of GSE24265 (Ritchie et al., 2015). Subsequently, by taking the intersection of the DEGs in our microarray assay, interesting genes in the key module, and the DEGs in GSE24265, candidate mRNAs were identified. GSE24265 was helpful in investigating the differences in gene expression between the perihematomal and normal brain tissues in ICH. The spatiotemporal expression patterns of the candidate mRNAs in the peripheral blood of ICH patients were analyzed using GSE125512. These results were visualized as Venn diagram and box plot using the “VennDiagram” and “ggplot 2” package in R.

### Functional Analysis of Key Module and Candidate mRNAs

Using the same method as above, GO terms and KEGG pathway enrichment analysis of the genes in the key module were performed. What is more, the samples of GSE24265 were grouped into a high-expressed group and a low-expressed group, according to the expression levels of single candidate mRNAs, using the R package “limma.” We further performed GSEA to explore the enriched function of candidate mRNAs with the R package “clusterProfiler” (Yu et al., 2012; Ritchie et al., 2015).

### Construction of a Protein–Protein Interaction Network

We built a protein–protein interaction (PPI) network with the STRING online database^2^ (Szklarczyk et al., 2019). Genes in the key module were imported into the STRING website, and we exported the PPI network data into a TSV file. Lastly, we used Cytoscape software to analyze and visualize the interaction network (Shannon et al., 2003). The “MCODE” plugin was utilized to explore the relationships between the different nodes in the network.

## Results

### Baseline Characteristics

In total, four male patients with ICH and four male VRF controls were recruited into our ceRNA microarray study. No significant differences in age distribution, hypertension, diabetes mellitus, hyperlipidemia, smoking status, and coronary artery disease were found between the ICH and control groups. Admission clinical data in the two groups, including systolic blood pressure, diastolic blood pressure, fasting blood glucose, and low-density lipoprotein (LDL), were not statistically different (Table 1). In the ICH group, hematomas were located in the putamen in three patients and in the thalamus in one patient. The mean hematoma volume was 25.41 ± 15.41 ml, which was assessed using the Coniglobus formula (Table 2; Tiwari et al., 2021).

**TABLE 1:** Demographic and clinical data of the subjects recruited in the microarray study.

**Variable**	**ICH group (*n* = 4)**	**Control group (*n* = 4)**	***p*-value**
Age, mean ± SD (years)	58.75 ± 9.29	60.00 ± 8.04	0.8456
Male, *n* (%)	4 (100)	4 (100)	1.0000
**Medical history, *n* (%)**			
Hypertension	4 (100)	4 (100)	1.0000
Diabetes mellitus	2 (50)	2 (50)	1.0000
Smoker	2 (50)	2 (50)	1.0000
Hyperlipidemia	2 (50)	2 (50)	1.0000
Coronary artery disease	0 (0)	0 (0)	1.0000
**Admission clinical data**			
Systolic blood pressure, mean ± SD (mmHg)	164.50 ± 23.97	144.50 ± 8.85	0.1961
Diastolic blood pressure, mean ± SD (mmHg)	98.75 ± 11.70	84.50 ± 12.87	0.1529
Fasting blood glucose, mean ± SD (mmol/L)	7.05 ± 1.76	7.62 ± 4.29	0.8164
LDL, mean ± SD (mmol/L)	3.04 ± 0.61	3.41 ± 0.80	0.4838

**ICH, intracerebral hemorrhage; LDL, low-density lipoprotein.**

**TABLE 2:** Location and volume of hematoma of the intracerebral hemorrhage (ICH) patients in the microarray study.

**Subjects**	**Location of hematoma**	**Volume of hematoma (ml)**
ICH 1	Putamen	13.83
ICH 2	Thalamus	14.04
ICH 3	Putamen	46.52
ICH 4	Putamen	27.26
Mean volume of hematoma, mean ± SD (ml)		25.41 ± 15.41
		

### DEGs in Peripheral Blood From Patients With ICH

**FIGURE 1:**
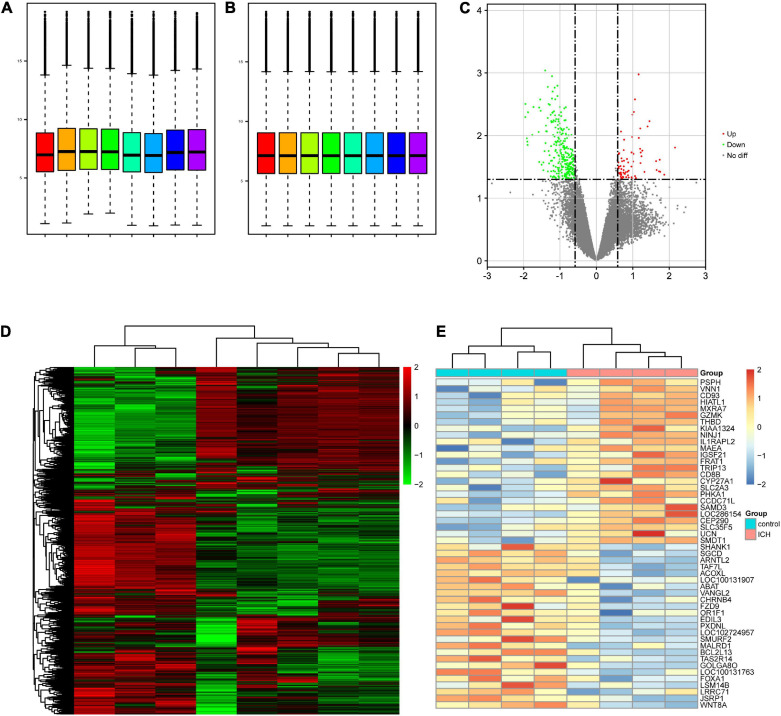
Differentially expressed genes (DEGs) present in the intracerebral hemorrhage (ICH) and control groups in our microarray. **(A)** Before normalization, there were background noise and measurement errors between the samples. **(B)** After normalization, the errors were eliminated. **(C)** Volcano plot showed the distribution of the DEGs between two groups. The *red* and *green dots* correspond to the upregulated and downregulated mRNAs, respectively. **(D)** Heatmap of the overall gene expression in our microarray. *Red* indicates the upregulated genes and *green* indicates the downregulated genes. **(E)** Heatmap of the top 25 upregulated DEGs (*red*) and the top 25 downregulated DEGs (*blue*) ranked by fold change. *No diff*, no difference.

After normalization, the mRNA expression levels of the samples were distributed at the same baseline (Figures 1A,B). A differential serum mRNA expression profile was found in the ICH group by setting the cutoff value as *p* < 0.05 and | fold change| > 1.5. Among the 13,566 mRNAs detected in our microarray, there were 340 DEGs between the ICH group and the control group, including 72 upregulated DEGs (PSPH, VNN1, CD93, HIATL1, MXRA7, among others) and 268 downregulated DEGs (WNT8A, JSRP1, LRRC71, LSM14B, FOXA1, among others). The distribution of the DEGs was visualized as volcano plot and hierarchical clustering heatmap plot (Figures 1C–E).

### Functional Enrichment Analysis of DEGs in Patients With ICH

**FIGURE 2:**
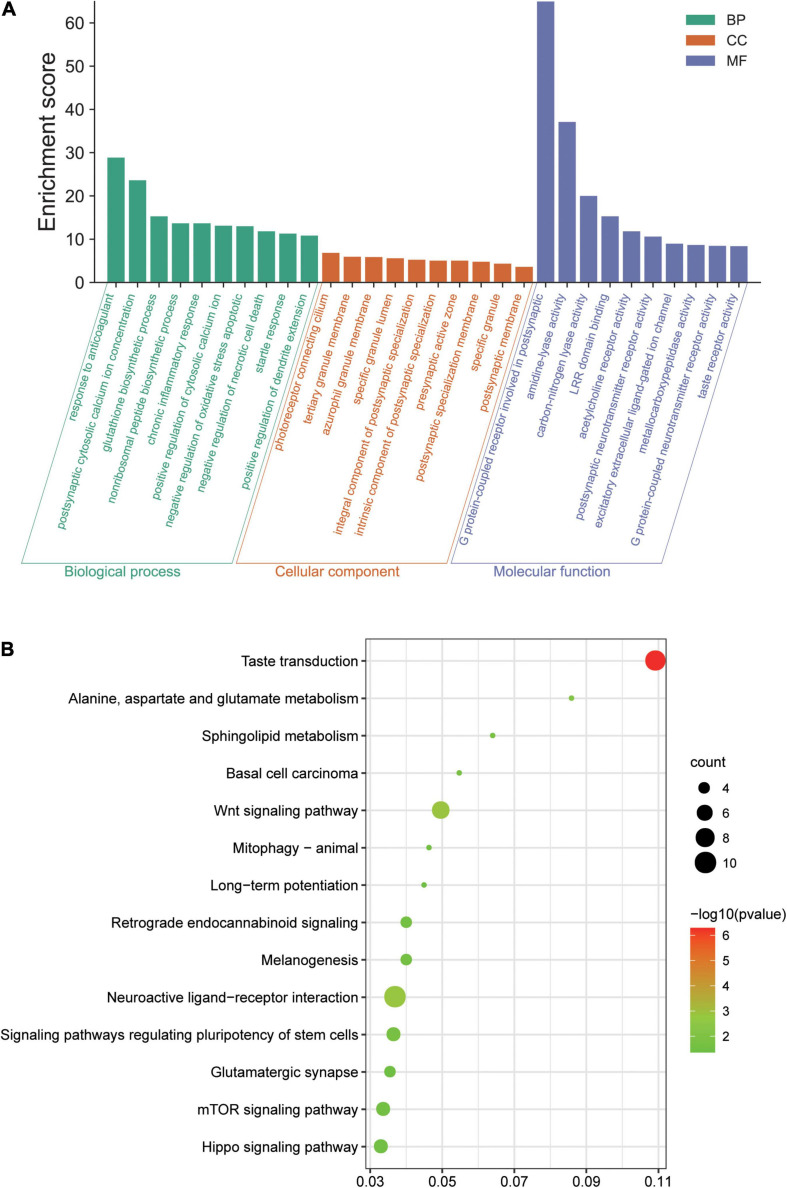
Results of the Gene Ontology (GO) terms and Kyoto Encyclopedia of Genes and Genomes (KEGG) pathway enrichment analysis of the differentially expressed genes (DEGs) in our array. **(A)** Top 10 terms in GO biological process (BP), cellular component (CC), and molecular function (MF) enrichment analysis, sorted by the enrichment score. **(B)** Significantly enriched KEGG pathways, gene count, and –log10 (*p-*value). *LRR*, leucine-rich repeat; *Wnt*, Wingless/integrated; *mTOR*, mammalian target of rapamycin.

GO and KEGG analyses were conducted involving all 340 DEGs. GO analysis suggested that, in the ICH group, the DEGs were mainly involved with negative regulation of necrotic cell death and chronic inflammatory response in terms of BP, that these genes were mostly enriched in the presynaptic active zone and postsynaptic membrane in terms of CC, and their MF focused on G protein-coupled neurotransmitter receptor activity and excitatory extracellular ligand-gated ion channel (Figure 2A). According to the KEGG pathway analysis, the genes were primarily enriched in pathways of Wnt signaling, the mammalian target of rapamycin (mTOR) signaling pathway, and neuroactive ligand–receptor interaction (Figure 2B).

### WGCNA and Identification of the Key Module

**FIGURE 3:**
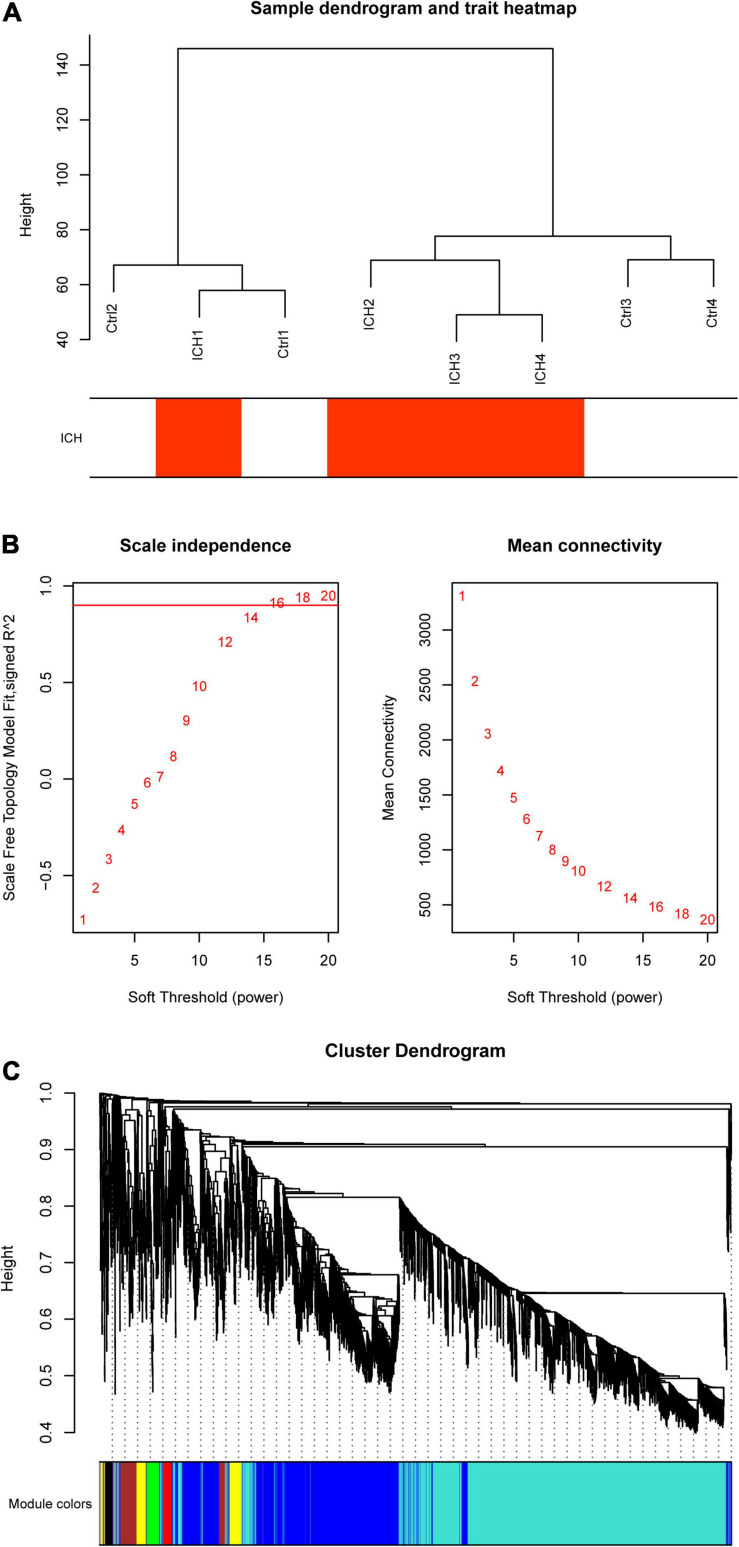
Co-expression network construction by weighted gene co-expression network analysis (WGCNA). **(A)** Sample data clustering showed no obvious outliers found. *Red color* represents the intracerebral hemorrhage (ICH) group. *Ctrl*, control group. **(B)** Determination of soft threshold. *Left*: Scale independence; *right*: Mean connectivity. **(C)** Cluster dendrogram. Each *color* represents one specific co-expression module; the *above branches* represent genes.

**FIGURE 4:**
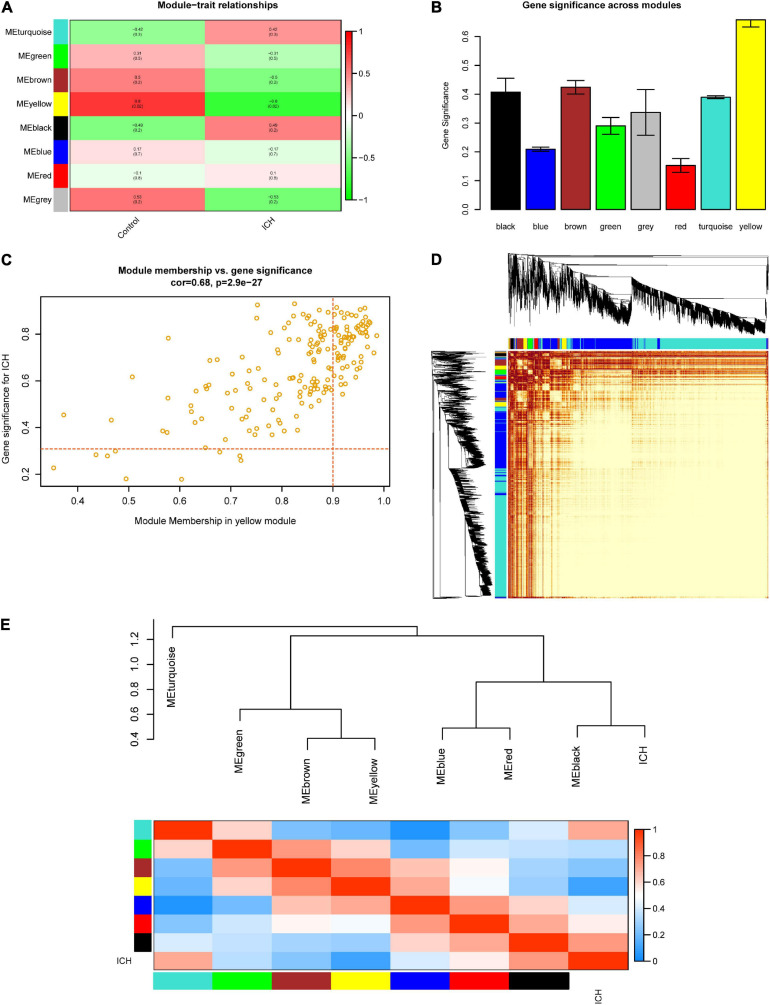
Selection of the key module and visualization of the topological overlap matrix (TOM). **(A)** Module–trait relationships among the eight gene modules. **(B)** Gene significance (GS) across modules. The GS value of the yellow module was the highest. **(C)** Module membership (MM) vs. GS in the yellow module. The *red dotted lines* represent the thresholds of MM > 0.9 and GS > 0.3 set for interesting genes. **(D)** Heatmap showing the TOM among all 5,000 genes involved in the WGCNA. **(E)** Eigengene dendrogram and adjacency heatmap further clarify the correlation between intracerebral hemorrhage (ICH) and the modules.

At the beginning, we selected the top 5,000 genes in our microarray assay by the method of MAD. Then, we clustered the sample data to see whether there were any obvious outliers; no outliers were found in our test (Figure 3A). We decided to set the soft thresholding power β to 16, bringing the scale-free topology *R*^2^-value to 0.9 (Figure 3B). The gene co-expression network was constructed with the one-step network construction method, and the genes were sorted into eight modules expressed by different colors (Figure 3C). The largest turquoise module contained 2,718 genes and the smallest gray module contained 27 genes. The module–trait relation and the GS across modules are shown in Figures 4A,B. The yellow module, whose GS was as high as 0.68 (*p* < 0.001), was chosen as the key module. Plots were charted to show the GS *vs*. MM in the yellow module, eigengene dendrogram, adjacency heatmap, and network heatmap (Figures 4C–E). Among the 191 genes in the yellow module, 68 interesting genes with MM > 0.9 and GS > 0.3 were singled out.

### Identification and Verification of Candidate mRNAs

**FIGURE 5:**
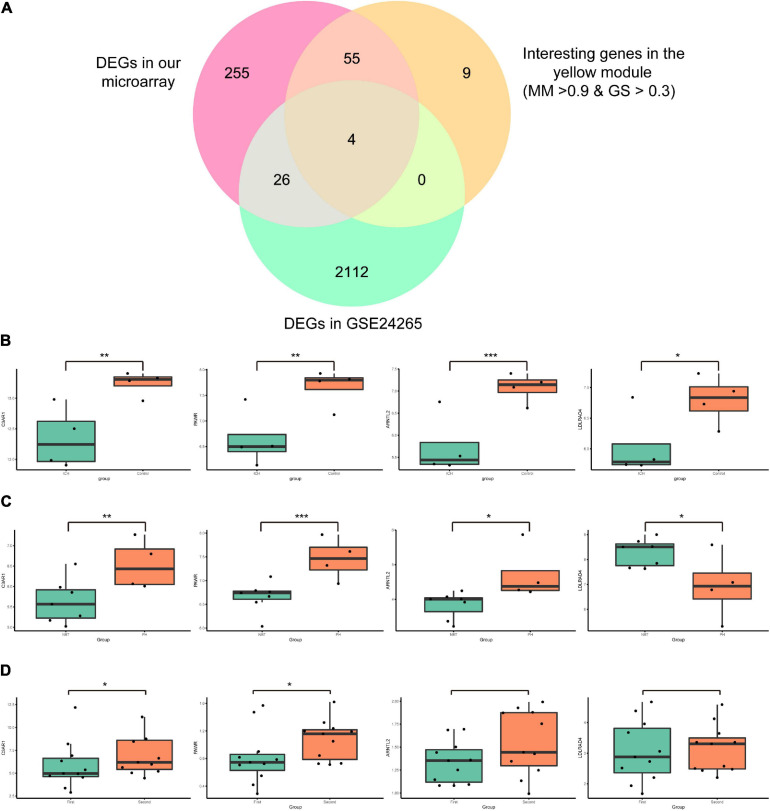
Identification and expressions of the candidate messenger RNAs (mRNAs) in our microarray and those of GSE24265 and GSE125512. **(A)** Venn diagram showing four candidate mRNAs in the intersection of the 340 differentially expressed genes (DEGs) in our array, 68 interesting genes in the yellow module, and the 2,142 DEGs in GSE24265. **(B)** Box plots of the expressions of the candidate mRNAs in our microarray data. The *green box* represents the intracerebral hemorrhage (ICH) group and the *red box* represents the control group. **(C)** Box plots of the expressions of candidate mRNAs in GSE24265. The *green box* represents the normal brain tissue (NBT) group and the *red box* represents the perihematomal tissue (PH) group. **(D)** Box plots of the expressions of candidate mRNAs in GSE125512. The *green box* represents the first blood collection group (blood was collected within 24 h of onset) and the *red box* represents the second blood collection group (blood was collected within 72 h after the first blood draw). ***p < 0.001; **p < 0.01; *p < 0.05.

Series matrix files of GSE24265 and GSE125512 were downloaded from the GEO website. The data were preprocessed in a similar manner to that above. In GSE24265, the difference analysis identified 2,142 DEGs (1,262 upregulated and 880 downregulated genes; *p* < 0.05 and | fold change| > 1.5). Four candidate mRNAs (C3AR1, PAWR, ARNTL2, and LDLRAD4) were screened out by taking the intersection of the 340 DEGs in our microarray data, 68 interesting genes in the yellow module, and 2,142 DEGs in GSE24265 (Figure 5A). In GSE125512, two of four candidate mRNAs (C3AR1 and PAWR) were also differentially expressed (*p* < 0.05). In the perihematomal areas, C3AR1, PAWR, and ARNTL2 were highly expressed, but LDLRAD4 had low expression. Within 24 h of onset of cerebral hemorrhage, all four candidate mRNAs (C3AR1, PAWR, ARNTL2, and LDLRAD4) were significantly downregulated in peripheral blood. After 72 ± 6 h, the expressions of C3AR1 and PAWR showed an increasing trend in peripheral blood (*p* < 0.05). However, the change of ARNTL2 and LDLRAD4 was not obvious. The expression patterns of the candidate mRNAs in our microarray study and two validation datasets are demonstrated in Figures 5B–D.

### Functional Analysis of the Key Module and Candidate mRNAs and PPI Network Construction

**FIGURE 6:**
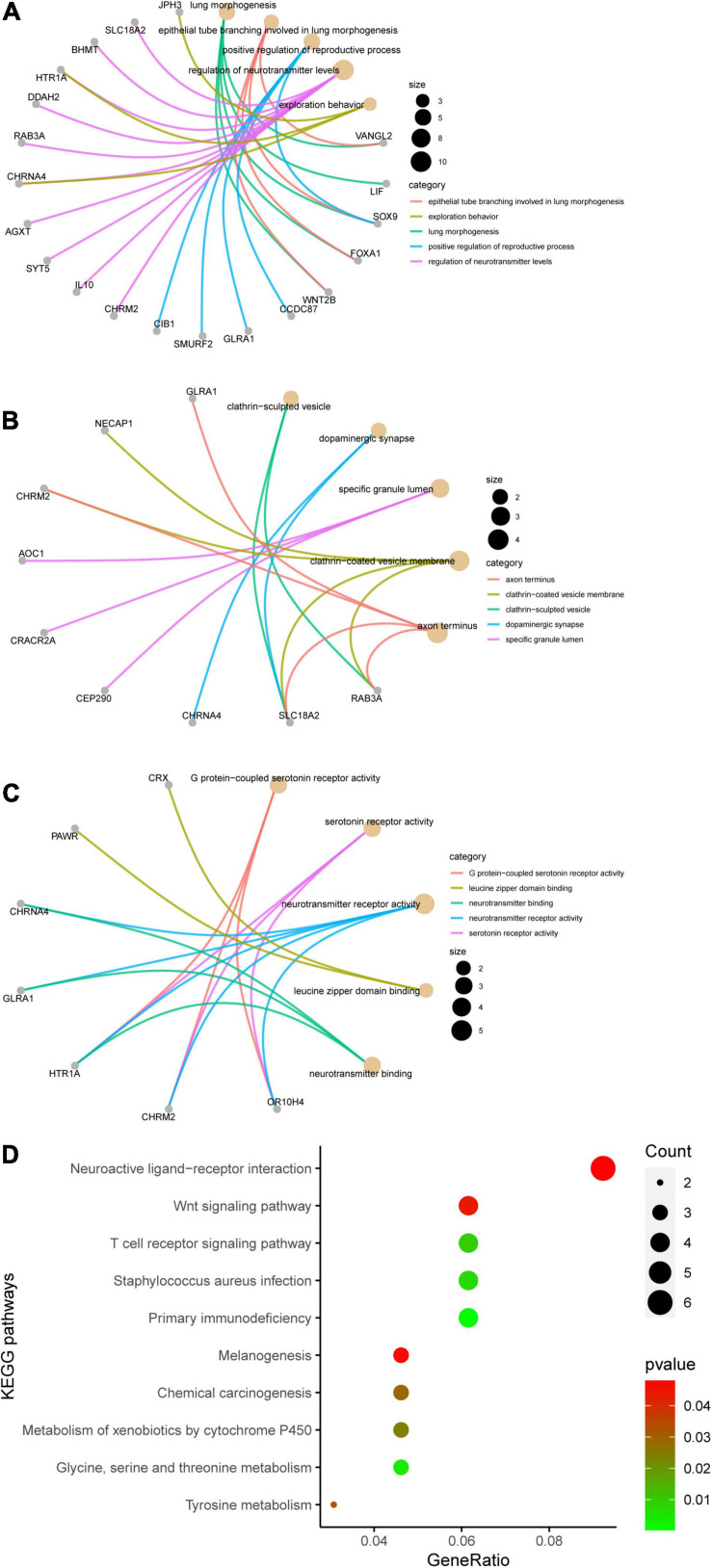
Results of the Gene Ontology (GO) terms and Kyoto Encyclopedia of Genes and Genomes (KEGG) pathway enrichment analysis of the genes in the yellow module. **(A)** Top 5 terms in GO biological process (BP). **(B)** Top 5 terms in GO cellular component (CC). **(C)** Top 5 terms in GO molecular function (MF). The *gray nodes* in **(A–C)** represent the genes related to the GO terms and the *golden nodes* represent the GO terms. **(D)** Top 10 enriched KEGG pathways, sorted by the gene ratio.

**FIGURE 7:**
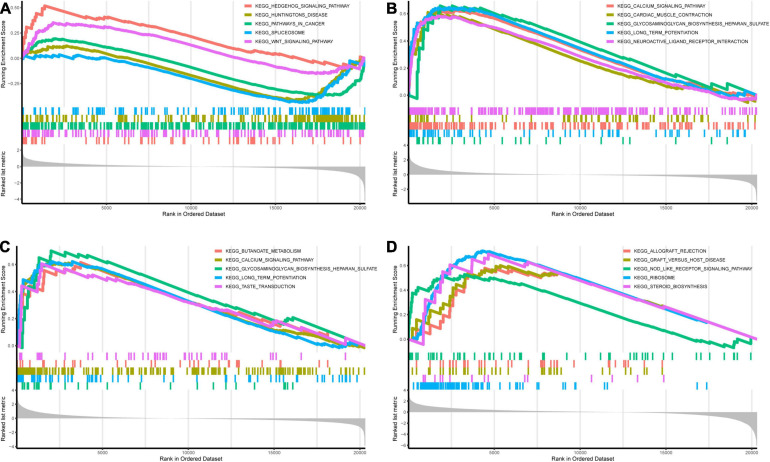
Gene set enrichment analysis (GSEA) of the candidate mRNAs in the GSE24265 dataset. **(A–D)** Top 5 gene sets (according to GSEA enrichment score) enriched in the high-expressed group of single candidate mRNAs. **(A)** *C3AR1*. **(B)** *PAWR*. **(C)** *ARNTL2*. **(D)** *LDLRAD4*.

**FIGURE 8:**
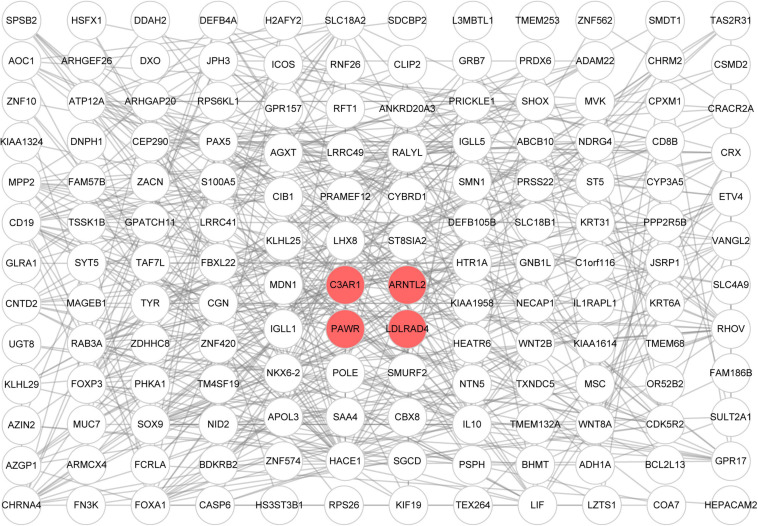
Construction of the protein–protein interaction (PPI) network consisting of genes from the yellow module. Each *circular node* represents a protein encoded by a gene, the *red nodes* are the candidate mRNAs, and the *line between nodes* represents the interaction between proteins.

Genes in the yellow module were enriched in GO biological process including regulation of neurotransmitter levels, GO cellular component including dopaminergic synapse, and GO molecular function including neurotransmitter receptor activity (Figures 6A–C). KEGG analysis showed that the Wnt signaling pathway and the neuroactive ligand–receptor interaction pathway were regulated by the genes in the yellow module (Figure 6D). According to the GSEA, C3AR1 might affect the Wnt signaling pathway, PAWR and ARNTL2 could act on the calcium signaling pathway, and the NOD-like receptor signaling pathway could be affected by the expression of LDLRAD4 in the brain tissues from ICH patients (Figure 7). With the help of STRING and Cytoscape software, the PPI network of the yellow module was constructed. The candidate mRNAs (C3AR1, PAWR, ARNTL2, and LDLRAD4, red colored) play crucial roles in the network (Figure 8).

## Discussion

Researchers have been trying to explore the blood mRNA profile of ICH. As early as 2015, Dykstra et al. used high-throughput sequencing to study the mRNA expression profiles of patients with cerebral hemorrhage. Among the differentiated genes, *INPP5D* (inositol polyphosphate-5-phosphatase) regulates myeloid cell proliferation and *ITA4* (integrin alpha 4) is involved in leukocyte recruitment in ICH (Dykstra-Aiello et al., 2015). In 2019, Stamova studied the peripheral blood transcriptome of patients with cerebral hemorrhage using GeneChip HTA 2.0 arrays. The study suggested that ICH had differentially expressed T cell receptor and CD36 genes and iNOS, TLR, macrophage, and T-helper pathways (Stamova et al., 2019). Two hundred and fifty changed mRNAs (136 upregulated and 114 downregulated), regulating many ICH-related pathways, such as the Toll-like receptor, natural killer cell, and TGF-β in the whole blood of patients with ICH (Cheng et al., 2020). GSE125512, which is the largest reported RNA-Seq study in ICH patients, has found significant changes in peripheral blood gene expression at 72–96 h compared with 0–24 h from ICH symptom onset. Many genes were related with the activation of the immune system and other inflammatory processes (Walsh et al., 2019).

However, there are a few studies about the correlation between RNA expression in human brain tissue and peripheral blood in ICH. GSE24265 showed the gene expressions in the brain tissues after ICH. The overexpressed genes in the perihematomal areas codify for cytokines, chemokines, coagulation factors, cell growth, and proliferation factors, while those underexpressed codify for proteins involved in cell cycle or neurotrophins (Rosell et al., 2011).

In the present study, 340 DEGs were identified in peripheral blood samples from patients with ICH by ceRNA microarray analysis, with functions enriched in many pathways, for instance, cell death, inflammatory response, and ligand-gated ion channel. These pathways have previously been reported to be associated with neuronal damage following ICH (Petzold et al., 2005; Zhu et al., 2012; Fu et al., 2018).

Through WGCNA, a total of eight gene co-expression modules were established. Among them, the yellow module was the main one related to ICH, containing 191 genes. These genes were enriched in multiple functional pathways, such as the Wnt signaling pathway, neurotransmitter receptor activity, and serotonin receptor activity. Undoubtedly, many researchers have reported that these pathways were involved in the pathogenesis of ICH (Liang et al., 2014; Zhou et al., 2014; Liu et al., 2020). Even more, selective serotonin reuptake inhibitors (SSRIs) were reported to have an unfavorable effect on the neurological outcomes of patients with ICH (Liu et al., 2020).

With the help of two GEO datasets (GSE24265 and GSE125512), we identified four candidate mRNAs (C3AR1, PAWR, ARNTL2, and LDLRAD4). It is worth mentioning that we have found different expression patterns of these mRNAs in peripheral blood and perihematomal tissues and that these changed with time. In the early stage of ICH (within 24 h), LDLRAD4 was found to have a low expression in perihematomal tissues; conversely, C3AR1, PAWR, and ARNTL2 were highly expressed. But all four genes showed low expressions in peripheral blood. However, in the late stage (72 h after the first blood draw), there was an obvious upward trend of C3AR1 and PAWR in peripheral blood. This suggested that C3AR1 and PAWR may play an important role in the progression of ICH.

*C3AR1* (complement C3a receptor 1) is a gene that encodes an orphan G protein-coupled receptor for C3a (Matsumoto et al., 2017). Previous studies have shown that the C3a level in the plasma of patients with aneurysmal subarachnoid hemorrhage (aSAH) correlated significantly with bad outcome, and the C3a receptor antagonist (C3aRA) could improve the neurologic outcome of experimental ICH in mice (Mack et al., 2007; Rynkowski et al., 2009). *PAWR* (pro-apoptotic WT1 regulator) is a pro-apoptotic gene that was among the top differentially expressed genes in peripheral blood mononuclear cells (PBMCs) from multiple sclerosis (MS) patients and might be involved in the regulation of MS (Comabella et al., 2016; Ghoveud et al., 2020). According to the GSEA in Figure 7, the expression of *C3AR1* could affect the Wnt signaling pathway, and the expression of *PAWR* had an effect on the calcium signaling pathway. The Wnt signaling pathway plays a role in the regulation of neuroinflammation and the calcium signaling pathway is involved in cell apoptosis (Zhang et al., 2019; Patergnani et al., 2020). Similar to what have been reported by a previous study, the overlap genes between the genes in the perihematomal brain tissue and those in peripheral blood were involved in neuroinflammation and apoptosis (Durocher et al., 2019).

Proteins encoded by the *ARNTL2* (aryl hydrocarbon receptor nuclear translocator like 2) gene are mostly relevant to circadian and hypoxia factors and are expressed in multiple regions of the brain (Hogenesch et al., 2000). GO annotations related to this gene include DNA-binding transcription factor activity. The low-density lipoprotein receptor class A domain containing 4 gene (LDLRAD4), previously named as *C18orf1*, could negatively regulate the transforming growth factor-β signaling and thereby probably plays a role in cell proliferation, differentiation, and apoptosis (Nakano et al., 2014). In this study, Figure 7 shows that, in patients with a high expression of *ARNTL2*, genes related to the calcium signaling pathway were enriched; in those with a high expression of *LDLRAD4*, genes related to the NOD-like receptor signaling pathway were enriched. As known, the NOD-like receptor signaling pathway has a significant influence on cell pyroptosis (Swanson et al., 2019). We could hypothesize that *ARNTL2* and *LDLRAD4* may be involved in neuronal apoptosis and pyroptosis in ICH, which needs to be explored in future studies.

Nevertheless, there are several limitations in our study. Firstly, the sample size for our ceRNA microarray study was insufficient, although we verified the expressions of the candidate mRNAs with GEO datasets. In future studies, a larger population of patients with ICH, and more peripheral blood and even brain tissue samples, is needed to verify the expressions of candidate mRNAs. Secondly, the exact influence mechanism of four candidate mRNAs on ICH is not clear. The possible mechanism of candidate mRNAs about neuroinflammation, neuronal apoptosis, and pyroptosis, indicated by functional analysis, would be the focus of our further studies.

Overall, four candidate mRNAs (C3AR1, PAWR, ARNTL2, and LDLRAD4) that may act notable roles in the pathophysiological process of ICH were identified. The influence mechanism of these mRNAs on ICH may be involved with neuroinflammation, cell apoptosis, pyroptosis, etc. We are expecting these mRNAs to be novel biomarkers or drug therapeutic targets for ICH and are looking forward to further studies on these mRNAs.

## Data Availability Statement

The original contributions presented in the study are included in the article/supplementary material, further inquiries can be directed to the corresponding author/s.

## Ethics Statement

The studies involving human participants were reviewed and approved by the Ethics Committee of The First Affiliated Hospital of China Medical University. The patients/participants provided their written informed consent to participate in this study.

## Author Contributions

FJ: data curation, formal analysis, methodology, software, and writing – original draft. LL: writing – review and editing and visualization. YH, LT, and YW: data curation and investigation. ZH: funding acquisition, conceptualization, supervision, and writing – review and editing. All authors contributed to the article and approved the submitted version.

## Conflict of Interest

The authors declare that the research was conducted in the absence of any commercial or financial relationships that could be construed as a potential conflict of interest.

## Footnotes

1 https://www.ncbi.nlm.nih.gov/geo/

2 https://string-db.org/
